# Racial and regional disparities of triple negative breast cancer incidence rates in the United States: An analysis of 2011–2019 NPCR and SEER incidence data

**DOI:** 10.3389/fpubh.2022.1058722

**Published:** 2022-12-01

**Authors:** Wei Zhang, Yuhui Bai, Caixing Sun, Zhangchun Lv, Shihua Wang

**Affiliations:** ^1^Department of Basic Medicine Sciences, Cancer Institute of The Second Affiliated Hospital, Zhejiang University School of Medicine, Hangzhou, China; ^2^Key Laboratory of Disease Proteomics of Zhejiang Province, Zhejiang University, Hangzhou, China; ^3^Shanghai Hongqiao International School, Shanghai, China; ^4^Department of Neurosurgery, The Cancer Hospital of the University of Chinese Academy of Sciences (Zhejiang Cancer Hospital), Institute of Basic Medicine and Cancer (IBMC), Chinese Academy of Sciences, Hangzhou, China; ^5^Key Laboratory of Head & Neck Cancer, Translational Research of Zhejiang Province, Hangzhou, China; ^6^Department of Medical Oncology, Yongkang Traditional Chinese Medicine Hospital, Yongkang, China; ^7^The Ohio State University Comprehensive Cancer Center - Arthur G. James Cancer Hospital and Richard J. Solove Research Institute, Columbus, OH, United States

**Keywords:** triple negative breast cancer, SEER, temporal trend, incidence, racial disparity, estrogen receptor, progesterone receptor, HER2

## Abstract

**Objective:**

Triple negative breast cancer (TNBC) is a more aggressive subtype resistant to conventional treatments with a poorer prognosis. This study was to update the status of TNBC and the temporal changes of its incidence rate in the US.

**Methods:**

Women diagnosed with breast cancer during 2011–2019 were obtained from the National Program of Cancer Registries (NPCR) and Surveillance, Epidemiology and End Results (SEER) Program SEER^*^Stat Database which covers the entire population of the US. The TNBC incidence and its temporal trends by race, age, region (state) and disease stage were determined during the period.

**Results:**

A total of 238,848 (or 8.8%) TNBC women were diagnosed during the study period. TNBC occurred disproportionally higher in women of Non-Hispanic Black, younger ages, with cancer at a distant stage or poorly/undifferentiated. The age adjusted incidence rate (AAIR) for TNBC in all races decreased from 14.8 per 100,000 in 2011 to 14.0 in 2019 (annual percentage change (APC) = −0.6, *P* = 0.024). Incidence rates of TNBC significantly decreased with APCs of −0.8 in Non-Hispanic White women, −1.3 in West and −0.7 in Northeastern regions. Women with TNBC at the age of 35–49, 50–59, and 60–69 years, and the disease at the regional stage displayed significantly decreased trends. Among state levels, Mississippi (20.6) and Louisiana (18.9) had the highest, while Utah (9.1) and Montana (9.6) had the lowest AAIRs in 2019. New Hampshire and Indiana had significant and highest decreases, while Louisiana and Arkansas had significant and largest increases in AAIR. In individual races, TNBC displayed disparities in temporal trends among age groups, regions and disease stages. Surprisingly, Non-Hispanic White and Hispanic TNBC women (0–34 years), and Non-Hispanic Black women (≥70 years) during the entire period, as well as Asian or Pacific Islander women in the South region had increased trends between 2011 and 2017.

**Conclusion:**

Our study demonstrates an overall decreased trend of TNBC incidence in the past decade. Its incidence displayed disparities among races, age groups, regions and disease stages. Special attention is needed for a heavy burden in Non-Hispanic Black and increased trends in certain groups.

## Introduction

Breast cancer is currently the most common female cancer and is continuously increasing in the US (1). Triple-negative breast cancer (TNBC) is one of the molecular subtypes without estrogen or progesterone receptors (ER or PR) and with low or no HER2 expression. This subtype is known to be more aggressive and unresponsive to conventional hormone therapy with a higher recurrence and worse survival (2–4). Characterizations of current TNBC status and temporal change are essential for exploring the underlying causes and for developing effective preventions and treatments.

TNBC has been reported to account for 8.4–15% of all breast cancers (5–10). The disease occurs most often in Black women (9, 11–13), proportionally higher in women at younger ages (13–17) and with tumors at a distant stage (6, 15). The incidence rate of breast cancer with ER and PR negative, but either positive or negative HER2, decreased by 1.5–2.6% per year in women aged ≥20 years in all racial/ethnic groups within the US during 2004–2016 (12). A population-based cross-sectional study reveals that TNBC incidence rates decreased in Non-Hispanic White (NHW) women aged 40 to 69 years and in Non-Hispanic Black (NHB) women aged 55–69 years from 2010 to 2016 (18). TNBC incidence rate displayed geographic variations correlated with socioeconomic status (10).

The recent national population-based study reports stark differences in age, race, and stage distribution at diagnosis of TNBC from other regional studies (6). Many previous findings are based on the data from local geographic areas and thus may not be generalizable well for the entire nation. Furthermore, temporal trends in TNBC incidence rates have not been updated among races, age groups and disease stages in more recent years. This study was conducted by utilizing the database covering the entire US population to study the status of TNBC and its temporal progression from 2011 to 2019.

## Patients and methods

Women diagnosed with breast cancer were obtained from the National Program of Cancer Registries and Surveillance, Epidemiology and End Results Program SEER^*^Stat Database: NPCR and SEER Incidence - US Cancer Statistics Public Use Research Database, 2021 Submission (2001–2019) covering 100% of the population for all 50 states and the District of Columbia in the US (19). TBNC was identified by “negative” in both “Merged estrogen receptor” and “Merged progesterone receptor,” as well as “HER2 negative, equivocal” in “Merged HER2 summary” in the database. Breast cancer with unknown statuses of ER, PR or HER2 was categorized as “Unknown.” All others were categorized as “non-TNBC.” Although the database began collecting the status of HER2 in 2010, only approximately 93% of the HER2 status was reported in breast cancer cases during that year. For that reason, this study only obtained and analyzed data during 2011–2019. Other variables were also included: age group, race, state, region, tumor differentiation, disease stage and surgery. Race/ethnicity was classified into NHW, NHB, Hispanic, Non-Hispanic Asian or Pacific Islander (API) and Non-Hispanic American Indian and Alaska Native (AIAN). Based on the “Merged Summary Stage” in the database, TNBC stage was grouped into localized, regional, distant and unknown. Tumor differentiation was categorized as well differentiated, moderately differentiated, poorly/undifferentiated and unknown. As the data are de-identified, human investigation approval was not necessary for this project.

## Statistical analysis

Categorical data was analyzed by the Chi Square test. We used SEER^*^Stat version 8.4.0 (National Cancer Institute) to calculate the age-adjusted incidence rates (AAIR) per 100,000 women by race, age, region and disease stage. The AAIR was age-adjusted to the 2000 US standard population. Tiwari et al.'s (20) modifications were applied to calculate the confidence interval (CI). The Joinpoint Regression Program (version 4.9.0.1, DigitCompass LLC, Maryland, USA) was used to generate incidence graphs and calculate the annual percent change (APC) using the least square method. For all analyses, *p* < 0.05 was deemed as statistically significant.

## Results

### Characteristic comparisons between TNBC and non-TNBC diagnosed during 2011-2019

From the year 2011 to 2019, a total of 2,725,735 breast cancers were diagnosed in women. Among them, 238,848 (or 8.8%) were TNBCs (Table 1). The proportions of TNBC in younger age groups were higher (3.5 vs. 1.4% for the 0–34 years age group and 20.2 vs. 16.5% for the 35–49 years age group) than those of non-TNBC. A significant racial disparity was observed for TNBC. As high as 21.1% of TNBC occurred in NHB women, which was in striking contrast with only 10.7% of non-TNBC in NHB women. The proportions of TNBC and non-TNBC were close in other races (Table 1). Regionally, the South had the highest TNBC cases (96,481 or 40.4%). There were significantly higher proportions of being poorly/undifferentiated (75.1 vs. 24.1%) or at a late distant stage (6.8 vs. 4.4%) in TNBC compared to non-TNBC (Table 1). A higher proportion of TNBC women received mastectomy surgery (42.9 vs. 34.7%) compared to that of non-TNBC women.

**Table 1:** Characteristics of newly diagnosed TNBC and Non-TNBC during 2011–2019.

**Variable**	**All Breast cancer *n* (%)**	**TNBC** ***n* (%)**	**Non-TNBC *n* (%)**	**Unknown** ***n* (%)**	***P* value**
	2,725,735 (100)	238,848 (8.8)	2,229,490 (81.8)	257,397 (9.4)	
**Age groups (years)**					
0–34	44,049 (1.6)	8,276 (3.5)	32,284 (1.4)	3,489 (1.4)	< 0.001
35–49	461,698 (16.9)	48,340 (20.2)	368,111 (16.5)	45,247 (17.6)	
50–59	632,306 (23.2)	59,508 (24.9)	511,223 (22.9)	61,575 (23.9)	
60–69	767,365 (28.2)	61080 (25.6)	641,603 (28.8)	64,682 (25.1)	
70 and over	820,317 (30.1)	61,644 (25.8)	676,269 (30.3)	82,404 (32)	
**Race**					
NHW	2,022,585 (74.2)	156,054 (65.3)	1,681,670 (75.4)	184,861 (71.8)	< 0.001
NHB	317,218 (11.6)	50,291 (21.1)	238,123 (10.7)	28,804 (11.2)	
Hispanic	231,276 (8.5)	21,683 (9.1)	183,792 (8.2)	25,801 (10)	
API	120,565 (4.4)	8,415 (3.5)	101,388 (4.5)	10,762 (4.2)	
AIAN	14,739 (0.5)	1,332 (0.6)	11,999 (0.5)	1,408 (0.5)	
Unknown	19,352 (0.7)	1,073 (0.4)	12,518 (0.6)	5,761 (2.2)	
**Region**					
Northeast	556,146 (20.4)	43,483 (18.2)	456,313 (20.5)	56,350 (21.9)	< 0.001
Midwest	594,293 (21.8)	53,422 (22.4)	497,616 (22.3)	43,255 (16.8)	
South	993,206 (36.4)	96,481 (40.4)	790,346 (35.4)	106,379 (41.3)	
West	582,090 (21.4)	45,462 (19)	485,215 (21.8)	51,413 (20)	
**Stage**					
Localized	1,429,088 (52.4)	144,060 (60.3)	1,241,172 (55.7)	43,856 (17.0)	< 0.001
Regional	587,373 (21.5)	72,930 (30.5)	499,763 (22.4)	14,680 (5.7)	
Distant	128,469 (4.7)	16,229 (6.8)	979,90 (4.4)	14,250 (5.5)	
Unknown	580,805 (21.3)	5,629 (2.4)	390,565 (17.5)	184,611 (71.7)	
**Differentiation**					
Well differentiated	504,582 (18.5)	4,536 (1.9)	482,513 (21.6)	17,533 (6.8)	< 0.001
Moderately differentiated	1,097,733 (40.3)	43,319 (18.1)	1,019,585 (45.7)	34,829 (13.5)	
Poorly/un-differentiated	770,431 (28.3)	179,331 (75.1)	537,854 (24.1)	53,246 (20.7)	
Unknown	352,989 (13.0)	11,662 (4.9)	189,538 (8.5)	151,789 (59)	
**Surgery**					
No surgery	278,847 (10.2)	30,136 (12.6)	196,077 (8.8)	52,634 (20.4)	< 0.001
Breast conserving surgery	1,457,218 (53.5)	106,559 (44.6)	1,246,413 (55.9)	104,246 (40.5)	
Mastectomy	942,305 (34.6)	100,522 (42.1)	773,332 (34.7)	68,451 (26.6)	
Unknown	47,365 (1.7)	1,631 (0.7)	13,668 (0.6)	32,066 (12.5)	

AIAN, American Indian/Alaska Native; API, Asian or Pacific Islander; NHB, Non-Hispanic Black; NHW, Non-Hispanic White; TNBC: Triple negative breast cancer.

### Temporal change of overall AACR for TNBC in all races/ethnicities during 2011-2019

**Figure 1:**
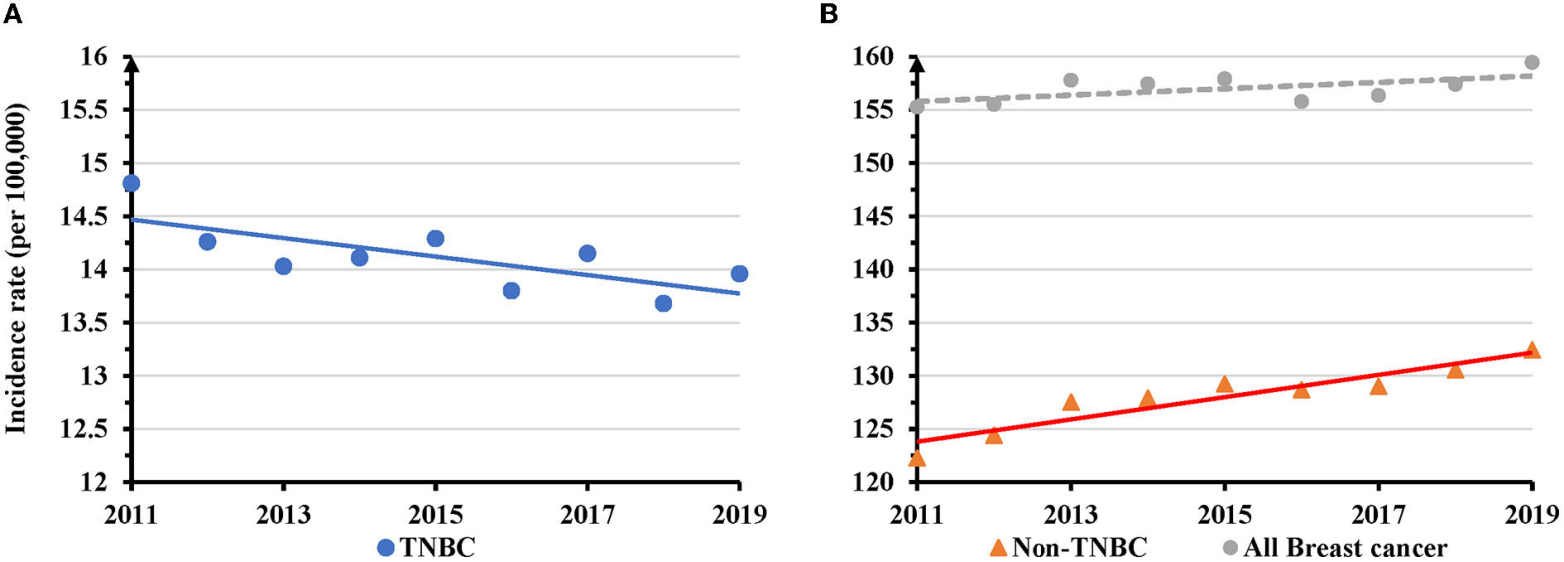
Temporal changes in overall age adjusted incidence rates of TNBC, non-TNBC and all breast cancer during 2011–2019. **(A)** TNBC displayed a decreased trend of annual age adjusted incidence rates (AAIRs). **(B)** Non-TNBC, but not all breast cancer, showed an increased trend of AAIRs. Solid trend lines indicate a significant change in APC, whereas dashed lines show a non-significant change.

The number of newly diagnosed TNBC cases slightly increased (4.3%), in contrast to non-TNBC (21.3%) or all breast cancer (14.3%) during 2011–2019 (Supplementary Table S1). The proportion of TNBC declined from 9.3 to 8.5% (Supplementary Figure S1). The overall AAIR for TNBC in women of all races/ethnicities showed a significant decrease from 14.8 per 100,000 women in 2011 to 14.0 in 2019. The annual percentage change (APC) was −0.6 (95% CI: −1.1 − −0.1, *P* = 0.024) during the study period (Figure 1A). Conversely, the AAIR for non-TNBC significantly increased from 122.3 per 100,000 women in 2011 to 132.4 in 2019 (APC = 0.3, 95% CI: 0.02–0.6, *P* = 0.041) (Figure 1B). The incidence rate of all breast cancer increased from 155.3 per 100,000 women in 2011 to 159.0 in 2019, but the trend was not significant (*P* = 0.090) (Figure 1B).

**Figure 2:**
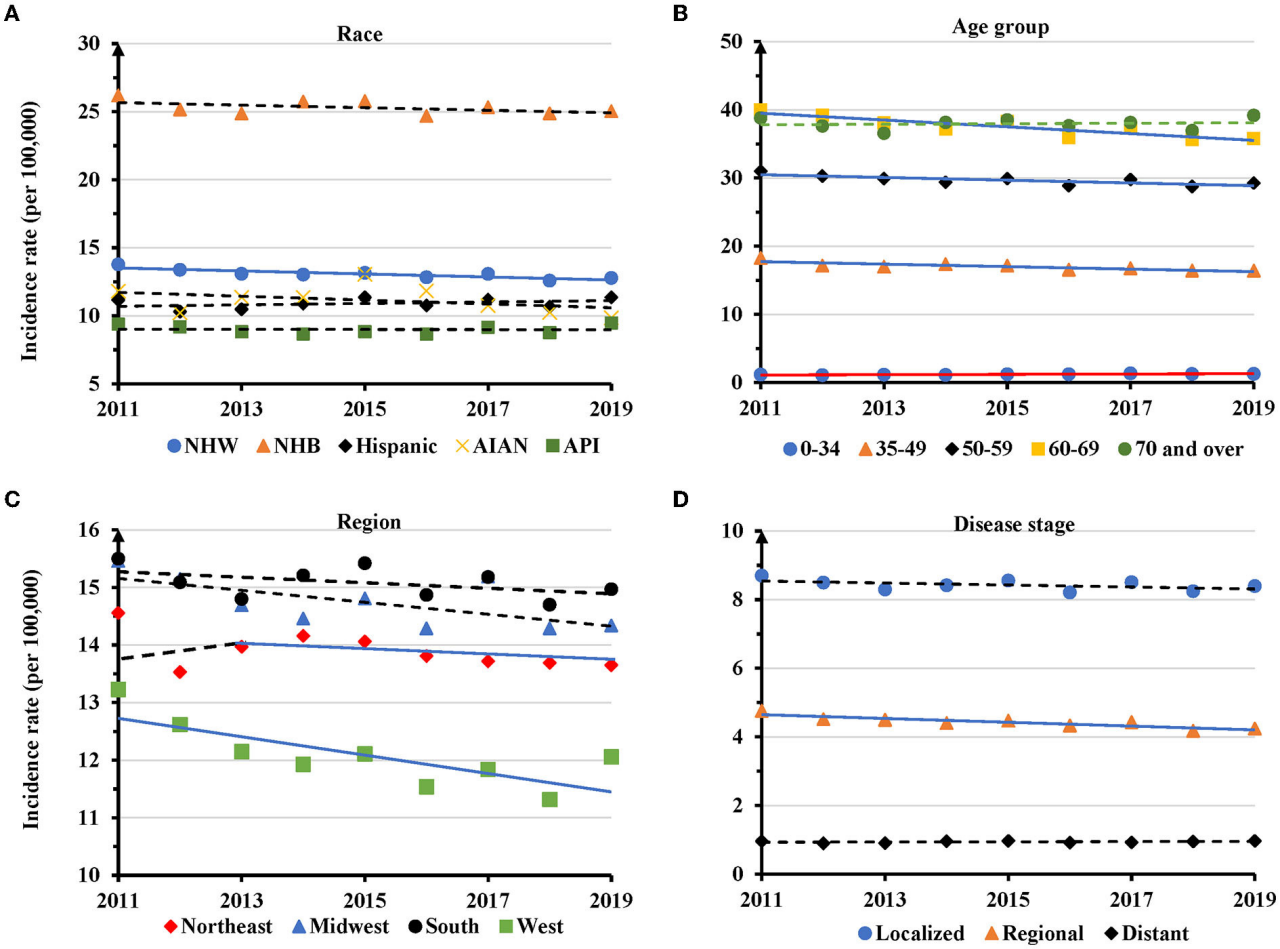
Temporal changes in age adjusted incidence rates of TNBC by race, age group, region and disease stage during 2011–2019. **(A)** Age group. **(B)** Race. **(C)** Region. **(D)** Disease stage. Solid lines indicate a significant annual percentage change (APC) of age adjusted incidence rate. Dashed lines show no significant change.

NHB women had the highest AAIR during the entire study period. In 2019, the AAIR was 25.0 per 100,000 women for NHB, but only 12.8 for NHW, 11.5 for Hispanic, 9.4 in AIAN and 9.5 in API (Figure 2A). There was a significantly decreased trend of AAIR with an APC of −0.8 (95% CI: −1.3 − −0.4, *P* = 0.003) in NHW, but not in other races (Figure 2A and Supplementary Table S2). Women at 35–49 (APC = −1.1, 95% CI: −1.6 − −0.5, *P* = 0.003), 50-59 (APC = −0.7, 95% CI: −1.1 − −0.2, *P* = 0.012), and 60–69 (APC = −1.3, 95% CI: −1.9 − −0.7, *P* = 0.001) years old displayed significantly decreased trends of AAIR. There was no significant change for women aged 70 years or older. Conversely, TNBC women aged 0–34 years displayed a significantly increased AAIR with an APC of 2.0 (95% of CI: 0.6–3.3, *P* = 0.010) (Figure 2B and Supplementary Table S2). Regionally, the South and Midwest regions had the highest AAIRs, while the West region showed the lowest AAIR for TNBC. The West region showed a significant decrease in AACR with an APC of −1.3 (95% CI: −2.2 − −0.3, *P* = 0.016) between 2011 and 2019, while the Northeastern region showed decreased AAIR with an APC of −0.4 (95% CI: −0.7 − −0.005, *P* = 0.048) only during 2013-2019 (Figure 2C and Supplementary Table S2). There was a significantly decreased trend of AAIR for TNBC diagnosed at the regional stage (APC = −0.6, 95% CI: −1.2 − −0.1, *P* = 0.021), but not for TNBC at a localized or distant stage (Figure 2D and Supplementary Table S2).

### Status and temporal changes of TNBC incidence in individual states

**Figure 3:**
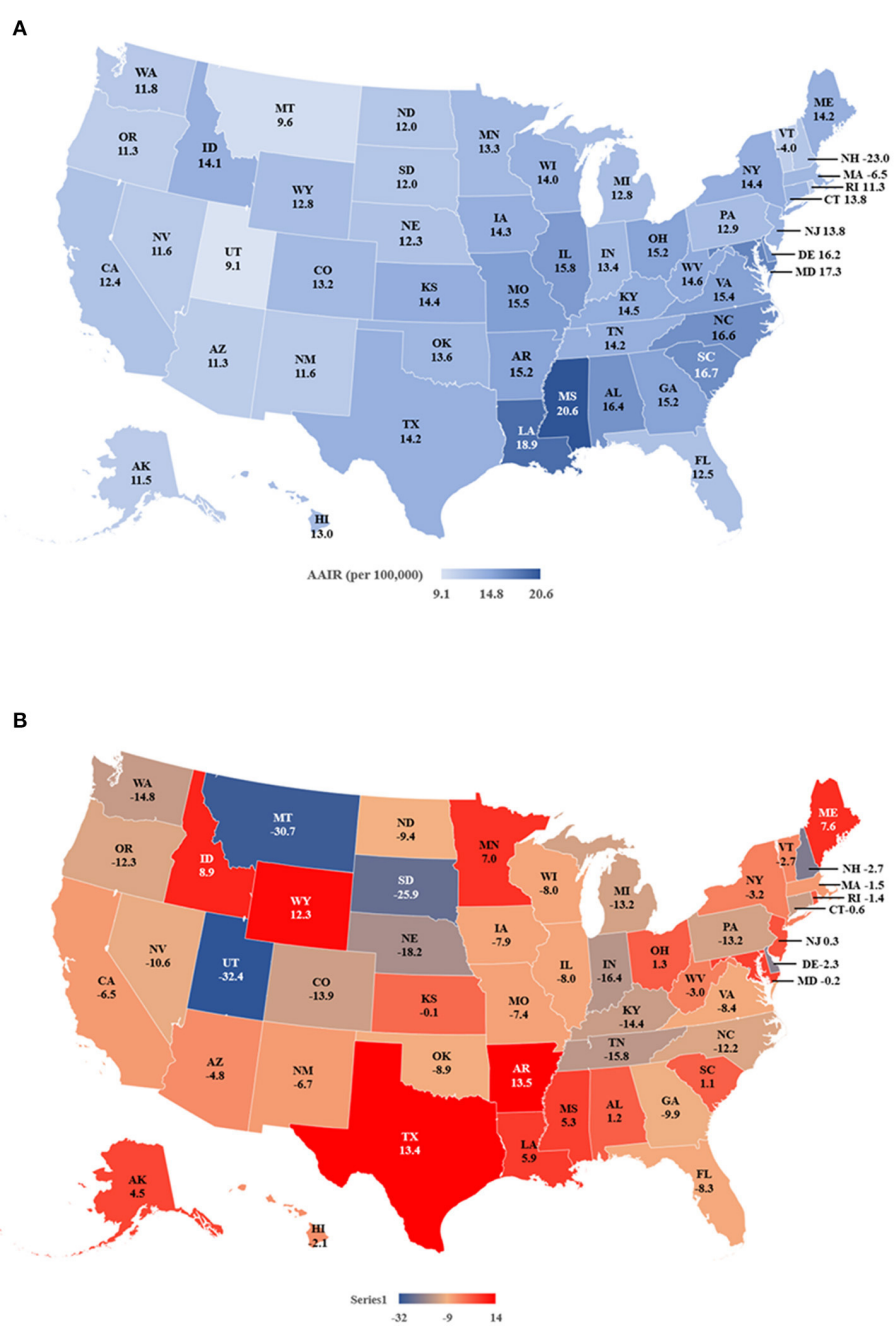
Age adjusted incidence rates (AAIR) of TNBC in individual states during 2011–2019. **(A)** AAIR of TNBC in 2019. **(B)** Percentage of change in AAIR in individual states from 2011 to 2019. Please note that there is no incidence rate of TNBC of NV in 2018 and 2019. The incidence rate of NV in 2017 is shown on the map.

The data indicated that during 2019 Mississippi (20.6 per 100,000 women), Louisiana (18.9), South Carolina (16.7) and North Carolina (16.6) had the highest AAIRs, while Utah (9.1), Montana (9.6) and Vermont (11.2) had the lowest (Figure 3A and Supplementary Table S3). Most states (34 of 50 states) showed decreased incidences from 2011 to 2019. Among them, Utah (−32.4%), Montana (−30.7%), and South Dakota (−25.9%) had the most significant percentage of decreases in AAIRs. In contrast, Arkansas (13.5%), Texas (13.4%) and Wyoming (12.3%) had the most percent increases (Figure 3B). Based on the APCs, New Hampshire (−2.7), Indiana (APC = −2.2) and Michigan (APC = −2.0) had the most significant decreases in AAIRs among 13 states, while Louisiana (1.9), Arkansas (1.5) and Texas (1.2) had significant increases in AAIR during the period (Supplementary Table S3).

### Comparison of the features of TNBC among individual races

Hispanic women with TNBC had a higher proportion diagnosed at younger age groups (6.4% in 0–34, and 32.4% in 35–49 years age groups), whereas NHW women had a higher proportion diagnosed at older ages (26.7% for 60–69, and 29.8% for 70 years or older) (Table 2). NHB women with TNBC had the highest proportion (59.0%) in the South region. NHB women had the highest proportion of TNBC (8.3%) diagnosed at the distant stage, and the highest proportion (79.2%) of being poorly/undifferentiated compared with women from other races/ethnicities. NHB women had the highest proportions of receiving no surgery (16.1%), but they had the lowest proportion (37.5%) of undergoing mastectomies (Table 2).

**Table 2:** Characteristics of newly diagnosed TNBC by races during 2011–2019.

	**NHW *n* (%)**	**NHB** ***n* (%)**	**Hispanic *n* (%)**	**API** ***n* (%)**	**AIAN *n* (%)**	***P* value**
	156,054 (65.6)	50,291 (21.1)	21,683 (9.1)	8,415 (3.5)	1,332 (0.6)	
**Age groups (years)**						
0–34	4,606 (3.0)	1,700 (3.4)	1,392 (6.4)	442 (5.3)	68 (5.1)	< 0.001
35–49	26,959 (17.3)	11,552 (23)	7,020 (32.4)	2,185 (26.0)	331 (24.8)	
50–59	36,346 (23.3)	14,794 (29.4)	5,577 (25.7)	2,171 (25.8)	373 (28)	
60–69	41,632 (26.7)	12,655 (25.2)	4,214 (19.4)	1,986 (23.6)	322 (24.2)	
70 and over	46,511 (29.8)	9,590 (19.1)	3,480 (16.0)	1,631 (19.4)	238 (17.9)	
**Region**						
Northeast	29,809 (19.1)	7,907 (15.7)	3,819 (17.6)	1,699 (20.2)	66 (5)	< 0.001
Midwest	41,561 (26.6)	8,931 (17.8)	1,605 (7.4)	867 (10.3)	229 (17.2)	
South	56,332 (36.1)	29,683 (59.0)	7,952 (36.7)	1,627 (19.3)	493 (37.0)	
West	28,352 (18.2)	3,770 (7.5)	8,307 (38.3)	4,222 (50.2)	544 (40.8)	
**Stages**						
Localized	97,951 (62.8)	27,558 (54.8)	12,112 (55.9)	5,072 (60.3)	776 (58.3)	< 0.001
Regional	44,438 (28.5)	17,558 (34.9)	7,577 (34.9)	2,618 (31.1)	416 (31.2)	
Distant	9,986 (6.4)	4,180 (8.3)	1,428 (6.6)	473 (5.6)	99 (7.4)	
Unknown	3,679 (2.4)	995 (2.0)	566 (2.6)	252 (3.0)	41 (3.1)	
**Differentiation**						
Well differentiated	3,357 (2.2)	649 (1.3)	309 (1.4)	184 (2.2)	20 (1.5)	< 0.001
Moderately differentiated	30,466 (19.5)	7,431 (14.8)	3,272 (15.1)	1,729 (20.5)	235 (17.6)	
Poorly/un- differentiated	114,826 (73.6)	39,815 (79.2)	16,820 (77.6)	6,084 (72.3)	997 (74.8)	
Unknown	7,405 (4.7)	2,396 (4.8)	1,282 (5.9)	418 (5.0)	80 (6.0)	
**Surgery**						
No surgery	17,285 (11.1)	8,097 (16.1)	3,341 (15.4)	1,036 (12.3)	167 (12.5)	< 0.001
Breast conserving surgery	70,612 (45.2)	23,002 (45.7)	8,656 (39.9)	3,280 (39)	575 (43.2)	
Mastectomy	67,175 (43)	18,877 (37.5)	9,486 (43.7)	4,030 (47.9)	580 (43.5)	
Unknown	982 (0.6)	315 (0.6)	200 (0.9)	69 (0.8)	10 (0.8)	

AIAN, American Indian/Alaska Native; API, Asian or Pacific Islander; NHB, Non-Hispanic Black; NHW, Non-Hispanic White; TNBC: Triple negative breast cancer.

### Temporal change of AAIR for TNBC in individual races during 2011–2019

**Figure 4:**
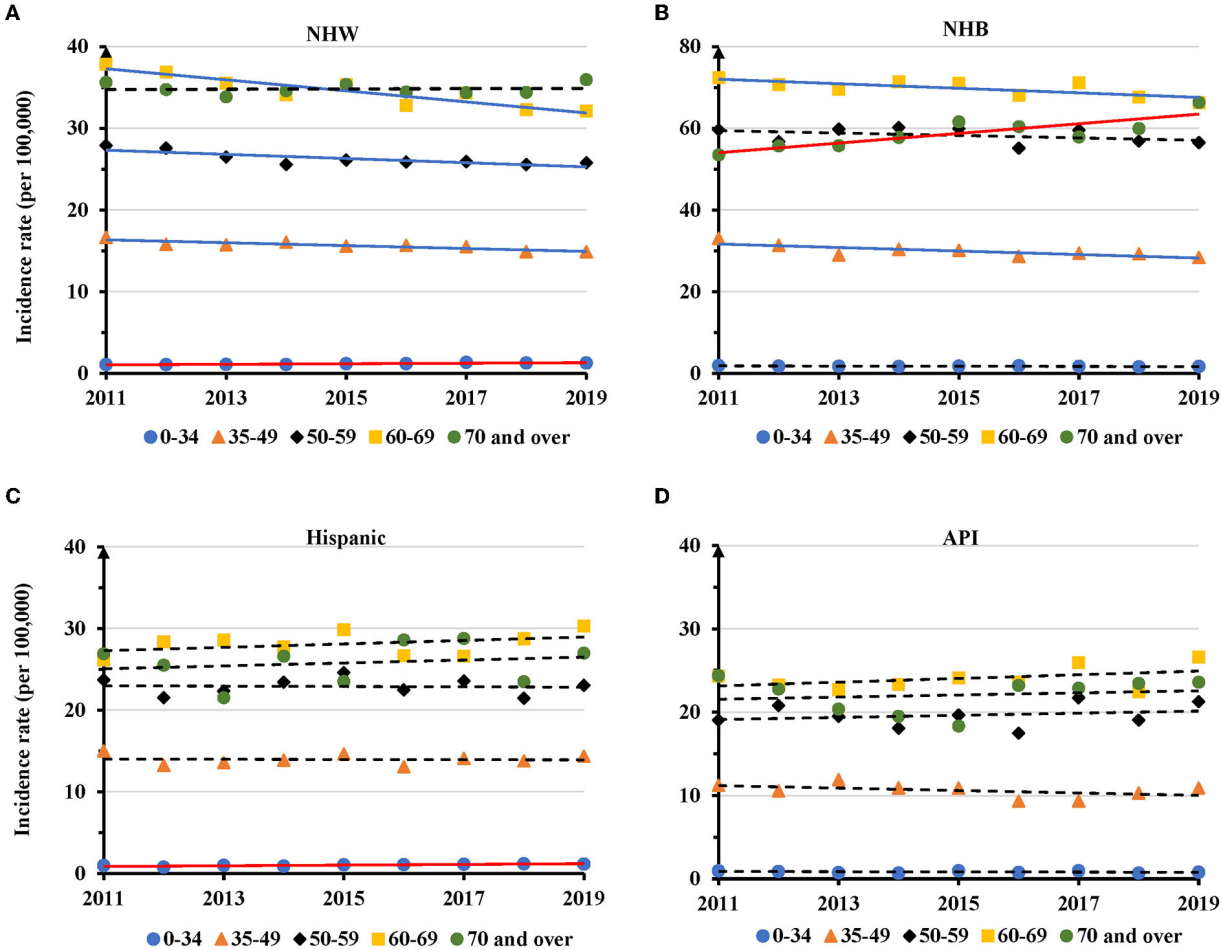
Temporal changes in age adjusted incidence rates of TNBC by age group in individual races during 2011–2019. **(A)** NHW. **(B)** NHB. **(C)** Hispanic. **(D)** API. Solid lines indicate significant change in annual percentage change (APC) of age adjusted incidence rate. Dashed lines show no significant change.

TNBC displayed disparities in temporal changes in different age groups of individual races. The AAIR showed a significantly increased trend in NHW women aged 0–34 years with an APC of 2.8 (95% CI: 1.7 − 4.0, *P* = 0.001), but decreased trends in the 35–49 (APC = −1.1, 95% CI: −1.6 − −0.6, *P* = 0.001), 50–59 (APC = −1.0, 95% CI: −1.6 − −0.4, *P* = 0.006), and 60–69 years age groups (APC = −1.9, 95% CI: −2.6 − −1.2, *P* < 0.001). No significant change was observed in NHW women aged 70 years and older (Figure 4A and Supplementary Table S4). For NHB women, there were significantly decreased AAIRs with an APC of −1.4 (95% CI: −2.4 − −0.5, *P* = 0.009) in 35–49, −0.8 (95% CI: −1.5 − −0.2, *P* = 0.021) in 60–69 age groups. NHB women at the age of 70 years and above had a significantly increased trend in AAIR with an APC of 2.1 (95% CI: 0.9–3.2, *P* = 0.004) during the period (Figure 4B and Supplementary Table S4). TNBC in Hispanic women aged 0–34 years had a significantly increased trend of AAIR (APC = 4.0, 95% CI: 1.5–6.7, *P* = 0.007). There was no significant change in APC in other age groups (Figure 4C and Supplementary Table S4). No significant change in AAIRs was found among different age groups in API women (Figure 4D and Supplementary Table S4). Since AIAN women had limited TNBC cases, their data was not presented.

**Figure 5:**
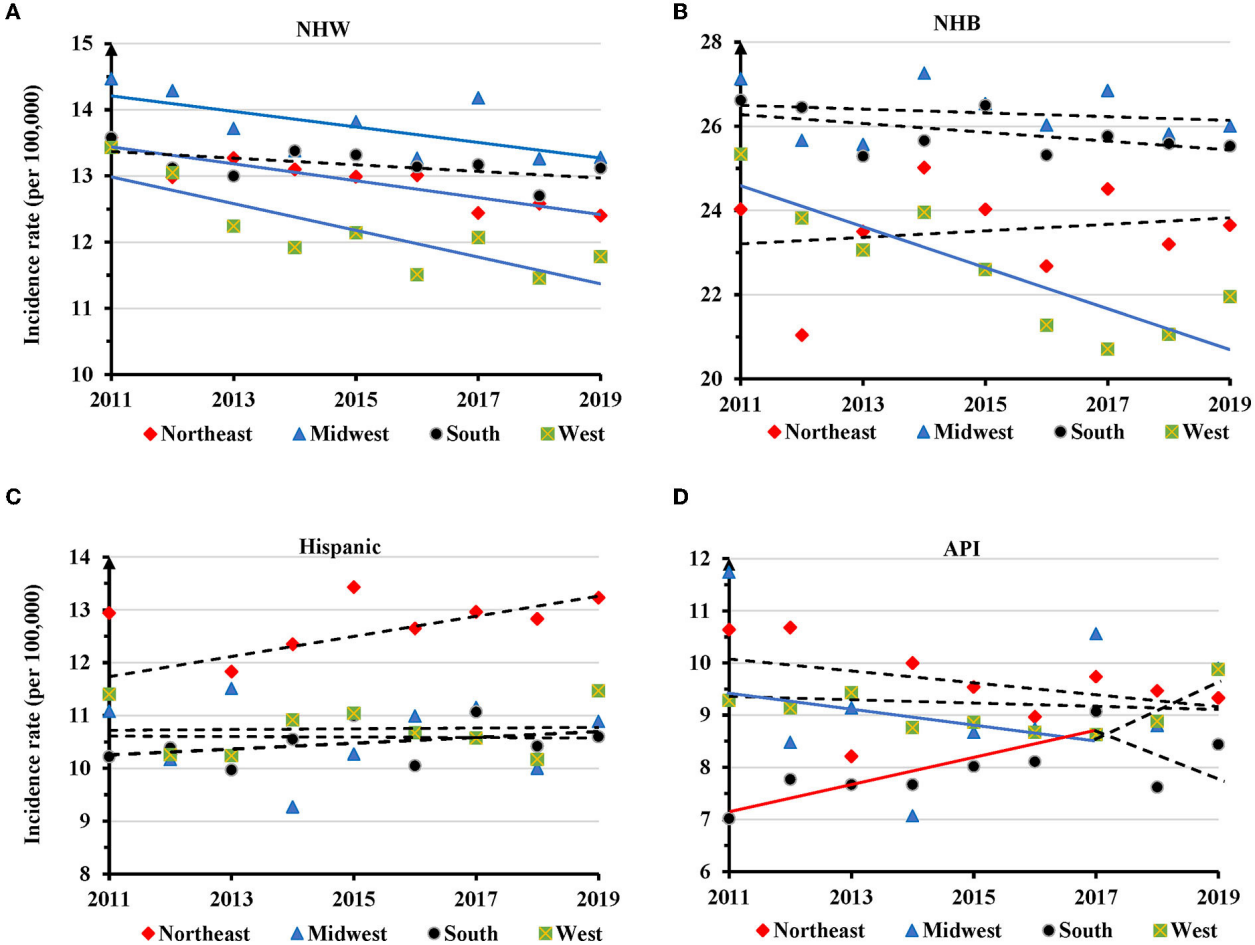
Temporal changes in age adjusted incidence rates of TNBC by region in individual races during 2011–2019. **(A)** NHW. **(B)** NHB. **(C)** Hispanic. **(D)** API. Solid trend lines indicate a significant annual percentage change (APC) of age adjusted incidence rate. Dashed lines show no significant change.

Regionally, the AAIRs for TNBC in NHW women had a significant decrease in the Northeast (APC=-1.0, 95% CI: −1.4 − −0.6, *P* = 0.001), Midwest (APC = −0.8, 95% CI: −1.7 − −0.02, *P* = 0.047) and West regions (APC = −1.7, 95% CI: −2.6 − −0.7, *P* = 0.005) (Figure 5A and Supplementary Table S5). NHB had a significant decrease of AAIR in the West (APC = −2.1, 95% CI: −3.2 − −1.0, *P* = 0.003) (Figure 5B and Supplementary Table S5). No significant change in TNBC incidence rate was observed in different regions in Hispanic women (Figure 5C and Supplementary Table S5). There was a decreased trend of AAIR for TNBC in API women in the West region with an APC of −1.5 (95% CI: −3.0 − −0.01, *P* = 0.047), but an increased trend in the South region with an APC of 2.8 (95% CI: 0.9–4.8, *P* = 0.014) only during the years of 2011–2017 (Figure 5D and Supplementary Table S5).

**Figure 6:**
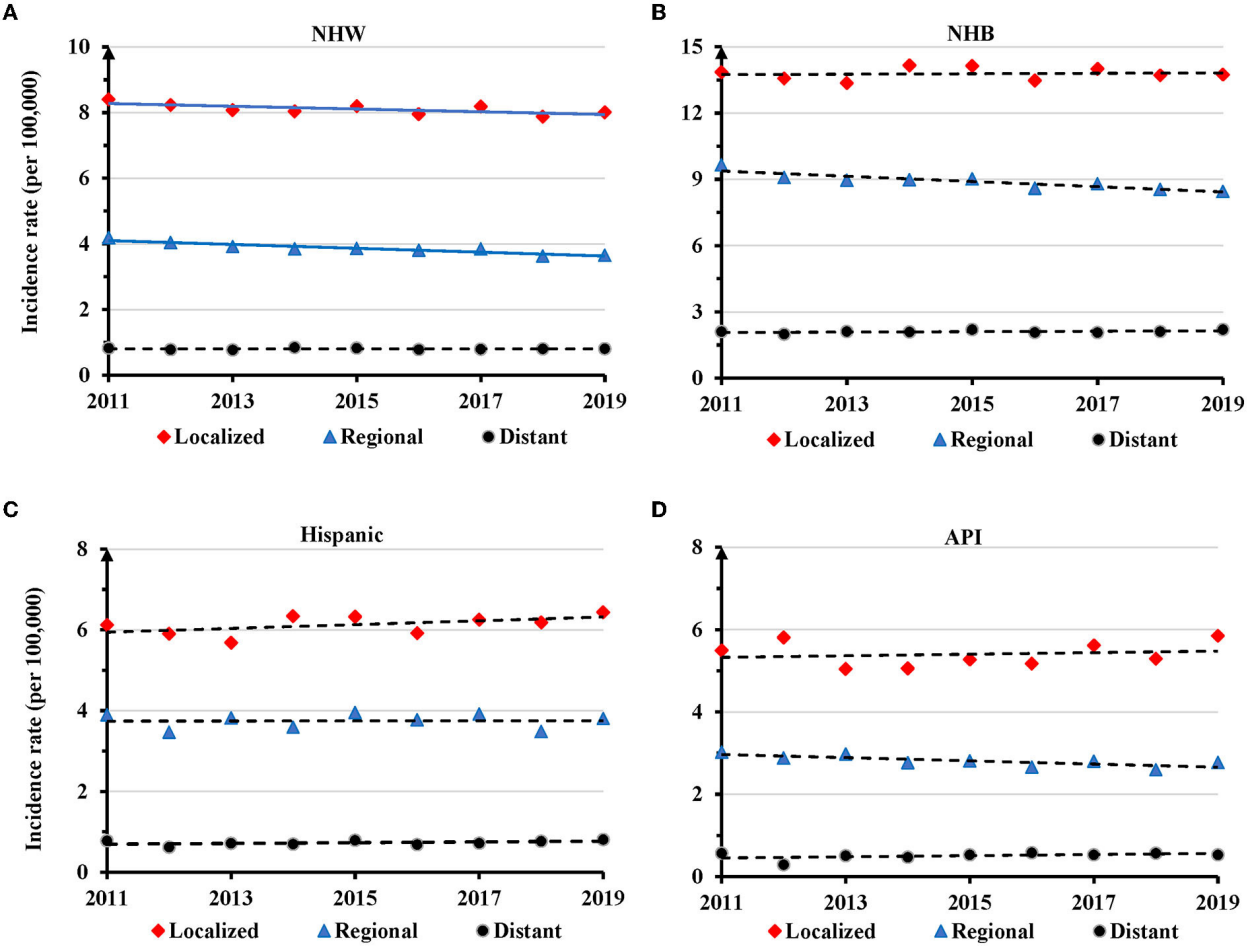
Temporal changes in age adjusted incidence rate of TNBC by disease stage in individual races during 2011–2019. **(A)** Non-Hispanic White (NHW). **(B)** Non-Hispanic Black (NHB). **(C)** Hispanic. **(D)** Asian or Pacific Islander (API). Solid lines indicate significant change in annual percentage change (APC) of age adjusted incidence rates. Dashed lines show no significant change.

There was a significant decrease in AAIR for TNBC at the localized (APC = −0.5, 95% CI: −1.0 − −0.1, *P* = 0.033) and regional (APC = −0.8, 95% CI: −1.3 − −0.4, *P* = 0.004) stages in NHW women during 2011-2019 (Figure 6A and Supplementary Table S6). There was no significant change in AAIR for TNBC at different disease stages in NHB, Hispanic, and API women (Figures 6B–D and Supplementary Table S6).

## Discussion

Using the NPCR and SEER database encompassing the entire population of the US, this study presented more generalizable updates on the TNBC incidence rate and its temporal trends during 2011–2019. We found that TNBC occurred disproportionally higher in women of NHB, younger ages, with cancer at a distant metastatic stage or being poorly/undifferentiated. The overall AAIR for TNBC in all races decreased during the study period. Only women of NHW, from West and Northeastern regions, women aged 35–49, 50–59, and 60–69 years old, or disease at a regional stage displayed significantly decreased trends. Among state levels, Mississippi and Louisiana had the highest, while Utah and Montana had the lowest AAIRs in 2019. New Hampshire and Indiana had the significant and highest decreases, Louisiana and Arkansas had the significant and largest increases in AAIR. In individual races, TNBC also displayed disparities in temporal trends among age groups, regions and disease stages. Surprisingly, increased trends were found in NHW and Hispanic TNBC women (0–34 years), and NHB women (≥70 years) during the entire period, as well as API women in the South region during 2011–2017.

Our results indicated that TNBC accounted for 8.8% of all breast cancer diagnoses during the period. This number is similar to 8.4% reported in a previous study (6), but is lower than 10–15% reported in many other previous studies (5–10). The decreased overall incidence of TNBC and the increased incidence rate of non-TNBC may explain the lower proportion of TNBC observed in this study. Our data revealed that NHB women had the highest AAIR for TNBC during the entire study period. In 2019, the AAIR in NHB women was 25.0 per 100,000 women in contrast to 12.6 or less in women of other races/ethnicities. NHB women aged 70 years or above displayed an increased trend of AAIR. NHB women accounted for as high as 21.1% of all TNBC, but only 10.7% of all non-TNBC. Women of other races/ethnicities had comparable proportions of TNBC and non-TNBC. Like this finding, approximately 21.0% TNBC cases (25,911 of 123,156 cases in all races/ethnicities) in NHB women were reported in one study (12), and 20.8% in another study (21). Further, this malignancy was proportionally higher in younger women as reported previously (13–17). The percentage (6.8%) of TNBC at the distant stage was comparable to results from previous studies (6, 22). This and other studies revealed that TNBC had a high percentage of being poorly/undifferentiated (9, 13, 23).

The temporal trend data indicated the overall incidence rate of TNBC in all races/ethnicities significantly decreased, and displayed disparities among races, age groups, regions, and disease stages during 2011–2019. The decreased trends of overall TNBC incidence were observed in NHW women, in women 35–69 years old, in West and Northeastern regions, and at the regional disease stage. Women 0–34 years old had an increased trend of AAIR for TNBC. A prior study utilizing the older version of the NPCR and SEER incidence database presented temporal changes in the incidence rate of hormone receptor (HR) (referred as ER and PR) positive and negative breast cancer in women aged 20 years or older during 2004–2016. Their results indicated an annual 1.5–2.6% decrease in the incidence rate of HR negative, but with both HER2 positive and negative, breast cancer among NHW, NHB, Hispanic, AIAN and API during the study period (12). Based on the SEER 13 database, another study indicated the change in TNBC incidence rates in NHW, NHB, Hispanic and API during 2010–2017 in their figure one without detailed information (24).

Further analyses uncovered the disparities of temporal trends in TNBC incidence among age groups in individual races. There was a significantly decreased trend of TNBC incidence in NHW women aged 35–69 years, and in NHB women aged 35–49 and 60–69 years. Our study found increased trends of TNBC incidence in NHW and Hispanic women aged 0–34 years, and NHB women in the age 70 years and above group. A previous study determined trends of incidence rates of TNBC and other molecular subtypes of breast cancer in women aged 25–80 years. Their results showed that TNBC incidence rates decreased annually in NHW women aged 40–54 years, and in both NHW and NHB women aged 55–69 years during 2010–2016 (18). It is noted that the SEER 18 cancer registry database which captures 27.8% of the US population, was utilized in their study. As described above, the data in 2010 only recorded the HER-2 status in 93% of breast cancer cases. Including the data of 2010 may bring a bias in the trend analysis. Using the database covering the entire population, our study disclosed more detailed trend disparities of TNBC incidence rates in dividual races by age groups within a longer and more recent study period.

One interesting finding is the regional and state disparities of TNBC incidence during the study period. The South region displayed the highest incidence rates and had the greatest number of new TNBC cases. Women in the West region had the lowest TNBC incidence rate, with significantly decreased trends in NHW and NHB. NHW women with TNBC also showed a decreased trend of AAIR in Northeast and Mideast regions. Conversely, API women showed a decreased trend of TNBC incidence in the West region, but an increased trend in the South region during 2011–2017. Mississippi, Louisiana, South Carolina and North Carolina had the highest AAIRs, while Utah, Vermont, and Montana had the lowest ones in 2019. Thirteen states showed significantly decreased trends, and 3 states displayed significantly increased trends of TNBC incidence rates. Moss et al. (10) calculated TNBC incidence rates for women of all races/ethnicities, and for their race or age subgroups in 43 states. Their results indicated that TNBC had the highest incidence rates in the South Atlantic and East South Central Census Divisions, and the lowest in the Mountain Division. Due to a short study period (2011–2013), no temporal change in TNBC incidence was presented.

Our study revealed that TNBC in NHW and Hispanic women aged 0–34 years displayed a trend of increased incidence during the study period. The proportion of TNBC in all breast cancer cases was the highest in this age group. Enhanced screening and diagnosis of breast cancer in these young women may possibly increase the incidence of TNBC. Young women diagnosed with TNBC often bear a family history, which is an important factor for mammogram screenings (25–27). A previous study estimated that 14.3% of women aged 18–39 years had a mammogram during 2011–2015 (28). Young women are reported to discover their own breast cancers more frequently by themselves (29). This study revealed a declined incidence of TNBC at localized and regional stages, and in older age groups (35–69 years) in NHW women. However, the incidence rates of TNBC in Hispanic women in older age groups were not significantly changed. Young NHB women had reported higher mammography utilization rates compared with women in other race/ethnicity groups (28, 30, 31), but the TNBC incidence rate of NHB women within this young age group showed no significant change. Early screening by mammography is generally not efficient in young women (25, 32). More studies are needed to explore the causes of the increased incidence of TNBC in NHW and Hispanic women at the young age group and to develop more effective preventive strategies for them.

Both biological risk and environmental factors may attribute to the high burden of TNBC in NHB women. High prevalence of BRCA1, BRCA2 and other genetic mutations in NHB women may lead to their increased susceptibility to TNBC (33–35). The TNBC incidence rate has been associated with state levels of sociodemographics, healthcare quality and health behaviors (10). Those in the South region often share lower socioeconomic status, worse health-related behaviors, and poorer health care (36, 37). This study showed that women in the South had a higher incidence rate of TNBC. Most NHB women with TNBC lived in the South region. Obesity has been associated with the risk of TNBC incidence in NHB (38, 39). The obesity epidemic has excessively affected NHB women (49.6% obese vs. 42.2% in NHW) in the US (40). This study revealed that NHB women had higher proportions of TNBC diagnosed at the distant stage or being poorly/undifferentiated. A higher proportion of NHB women received no surgical treatment. These findings imply that NHBs have reduced access to timely and high-quality health care related to prevention, early detection, and treatment services (41–44). These more aggressive pathological features are correlated with the worse prognosis of TNBC reported in NHB women (45). Many other non-biologic factors may contribute to the higher incidence and more aggression of TNBC in NHB women (46). Ethnic-specific strategies are needed to curb the high burden of TNBC in NHB women.

This study showed the decreased overall trend of TNBC incidence in all races/ethnicities, and the decreased trend in certain age groups, regions and disease stages of individual races during the study period. Based on the PR, ER, and HER2 statuses, breast cancer can be divided into four molecular subtypes: TNBC, luminal A, luminal B and HER2 enriched (9). This study showed increased incidence rates of non-TNBC in women of all races/ethnicities between 2011 and 2019. Similarly, the increased trend of non-TNBC incidence has been reported previously during 2010–2016 (18). Both biological and non-biological risk factors have been attributed to the development of breast cancer (47–52). The opposite trends between incidence rates of TNBC and non-TNBC emphasize clinical significance to explore their unique underlying mechanisms.

There are certain limitations in our current population based study. Most of our findings are descriptive due to the lack of individual case information in the database. Since no individual age year was available in the database, the AACR is calculated based on the 5-year age group. Many tumor clinicopathological data were not available, such as tumor size, T, N and M stages, and treatments other than surgery. No survival information was available in this database. Demographic and socioeconomic factors, such as employment, health insurance, household income, marital status and education, were not included. This database lacks information on family history or genetic test results. Due to data of limited years, the trend analyses were limited to one or the most two points. The strength of this study is that we utilize the thus far most updated database covering the entire population of the US, which allows us to disclose the disparities among ages, races, regions (states), and disease stages.

## Conclusions

TNBC compromises 8.8% of breast cancer cases and displays a trend of a decreased incidence rate during the past decade. The incidence rate and its temporal change display disparities among age, race, region and disease stage. More attention needs to be paid to the heavy burden of TNBC in NHB and increased trends in certain groups.

## Data availability statement

The original contributions presented in the study are included in the article/Supplementary material, further inquiries can be directed to the corresponding authors.

## Ethics statement

Ethical review and approval was not required for the study on human participants in accordance with the local legislation and institutional requirements. Written informed consent for participation was not required for this study in accordance with the national legislation and the institutional requirements.

## Author contributions

ZL and SW were responsible for the original concept of the study. WZ and CS designed the study. YB, WZ, and SW were responsible for data collecting, processing, and analysis. WZ, SW, ZL, CS, and YB drafted the manuscript. All authors have read and approved the final manuscript.

## Funding

This study was funded by National Science Foundation of Zhejiang Province (Grant Numbers: LY21H160007 and LY22H160043).

## Conflict of interest

The authors declare that the research was conducted in the absence of any commercial or financial relationships that could be construed as a potential conflict of interest.

## Publisher's note

All claims expressed in this article are solely those of the authors and do not necessarily represent those of their affiliated organizations, or those of the publisher, the editors and the reviewers. Any product that may be evaluated in this article, or claim that may be made by its manufacturer, is not guaranteed or endorsed by the publisher.

## Abbreviations

AAIR: age adjusted incidence rate
AIAN: American Indian/Alaska Native
APC: annual percentage change
API: Asian or Pacific Islander
ER: estrogen receptor
HER2: human epidermal growth factor receptor 2
NHB: Non-Hispanic Black
NHW: Non-Hispanic White
NPCR: National Program of Cancer Registries
PR: progesterone receptor
SEER, Surveillance: Epidemiology and End Results Program
TNBC: triple negative breast cancer.
