# Supplementary material for: Racial and regional disparities of triple negative breast cancer incidence rates in the United States: An analysis of 2011–2019 NPCR and SEER incidence data
Source: Front Public Health. 2022 Dec 1;10:1058722. doi: 10.3389/fpubh.2022.1058722 (PMC9752091; doi:10.3389/fpubh.2022.1058722)
Supplement: Supplementary file 1 [file Table_1.docx]

**Supplemental data**

Table S1. Number of TNBC and non-TNBC cases during 2011-2019

|  | All Breast cancer  n (%) | TNBC  n (%) | Non-TNBC  n (%) | Unknown  n (%) |
| --- | --- | --- | --- | --- |
|  | 2,725,735 (100) | 238,848 (8.8) | 229,490 (81.8) | 257,397 (9.4) |
| Year |  |  |  |  |
| 2011 | 281,889 (10.3) | 26,337 (11.0) | 2,22260 (10.0) | 33,292 (12.9) |
| 2012 | 286,808 (10.5) | 25,785 (10.8) | 229,790 (10.3) | 31,233 (12.1) |
| 2013 | 295,459 (10.8) | 25,728 (10.8) | 239,170 (10.7) | 30,561 (11.9) |
| 2014 | 299,623 (11.0) | 26,193 (11) | 243,930 (10.9) | 29,500 (11.5) |
| 2015 | 305,505 (11.2) | 26,985 (11.3) | 250,563 (11.2) | 27,957 (10.9) |
| 2016 | 306,090 (11.2) | 26,367 (11) | 253,602 (11.4) | 26,121 (10.1) |
| 2017 | 311,825 (11.4) | 27,428 (11.5) | 258,219 (11.6) | 26,178 (10.2) |
| 2018 | 315,104 (11.6) | 26,558 (11.1) | 262,277 (11.8) | 26,269 (10.2) |
| 2019 | 323,432 (11.9) | 2,7467 (11.5) | 269,679 (12.1) | 26,286 (10.2) |
| Change from 2011-2019 (%) | 14.7 | 4.3 | 21.3 | -21.0 |

Abbreviation: TNBC, triple negative breast cancer.

Table S2. Joinpoint analysis of trends of TNBC incidence rate by race, age group and disease stages.

| Variable | Trend 1 | APC | 95% CI | P value | Trend 2 | APC | 95% CI | *P* value |
| --- | --- | --- | --- | --- | --- | --- | --- | --- |
| Race |  |  |  |  |  |  |  |  |
| NHW | 2011-2019 | -0.8* | -1.3 - -0.4 | 0.003 |  |  |  |  |
| NHB | 2011-2019 | -0.4 | -0.9 - 0.2 | 0.157 |  |  |  |  |
| Hispanic | 2011-2019 | 0.5 | -0.6 - 1.5 | 0.308 |  |  |  |  |
| AIAN | 2011-2019 | -1.4 | -4.0-1.4 | 0.280 |  |  |  |  |
| API | 2011-2019 | 0.03 | -1.1-1.2 | 0.944 |  |  |  |  |
|  |  |  |  |  |  |  |  |  |
| Age group (years) |  |  |  |  |  |  |  |  |
| 0-34 | 2011-2019 | 2.0* | 0.6 - 3.3 | 0.010 |  |  |  |  |
| 35-49 | 2011-2019 | -1.1* | -1.6 - -0.5 | 0.003 |  |  |  |  |
| 50-59 | 2011-2019 | -0.7* | -1.1 - -0.2 | 0.012 |  |  |  |  |
| 60-69 | 2011-2019 | -1.3* | -1.9 - -0.7 | 0.001 |  |  |  |  |
| 70 and over | 2011-2019 | 0.4 | -0.3 - 1.1 | 0.233 |  |  |  |  |
|  |  |  |  |  |  |  |  |  |
| Region |  |  |  |  |  |  |  |  |
| Northwest | 2011-2013 | -1.1 | -1.1 - 1.0 | 0.223 | 2013-2019 | -0.4* | -0.7 - -0.005 | 0.048 |
| Midwest | 2011-2019 | -0.7 | -1.4 - 0.02 | 0.054 |  |  |  |  |
| South | 2011-2019 | -0.3 | -0.8 - 0.2 | 0.192 |  |  |  |  |
| West | 2011-2019 | -1.3* | -2.2 - 0.3 | 0.016 |  |  |  |  |
|  |  |  |  |  |  |  |  |  |
| Stage |  |  |  |  |  |  |  |  |
| Localized | 2011-2019 | -0.3 | -0.9- 0.2 | 0.162 |  |  |  |  |
| Regional | 2011-2019 | -0.6* | -1.2 - -0.1 | 0.021 |  |  |  |  |
| Distant | 2011-2019 | 0.3 | -0.5 - 1.2 | 0.413 |  |  |  |  |

Abbreviations: AIAN, American Indian/Alaska Native; APC, annual percentage change; API, Asian or Pacific Islander; NHB, Non-Hispanic black; NHW, Non-Hispanic white; TNBC, Triple negative breast cancer.

* indicates a significant change.

Table S3. Age adjusted incidence rates of TNBC in individual states during 2011-2019.

|  |  | Year | | | | | | | |  |  |
| --- | --- | --- | --- | --- | --- | --- | --- | --- | --- | --- | --- |
| State | 2011 | 2012 | 2013 | 2014 | 2015 | 2016 | 2017 | 2018 | 2019 | 2011-19 APC | PC |
| Alabama | 16.2 | 13.1 | 15.1 | 17.6 | 16.7 | 16.4 | 17.4 | 15.2 | 16.4 | 1.0 | 1.2 |
| Alaska | 11.0 | 16.4 | 9.6 | 17.0 | 11.6 | 14.1 | 14.4 | 10.6 | 11.5 | -1.4 | 4.5 |
| Arizona | 11.9 | 11.6 | 10.2 | 11.0 | 12.0 | 12.0 | 11.7 | 10.4 | 11.3 | -0.3 | -4.8 |
| Arkansas | 13.4 | 14.1 | 14.7 | 14.5 | 16.2 | 15.1 | 15.3 | 15.6 | 15.2 | 1.50* | 13.5 |
| California | 13.2 | 12.6 | 12.7 | 12.3 | 12.2 | 11.3 | 11.9 | 11.5 | 12.4 | -1.26* | -6.5 |
| Colorado | 15.4 | 13.7 | 14.4 | 12.5 | 13.0 | 12.4 | 13.0 | 12.3 | 13.2 | -1.91* | -13.9 |
| Connecticut | 16.1 | 13.5 | 14.1 | 15.1 | 15.0 | 13.9 | 15.2 | 14.5 | 13.8 | -0.6 | -14.3 |
| Delaware | 20.7 | 18.9 | 17.0 | 19.2 | 21.0 | 18.2 | 18.0 | 15.8 | 16.2 | -2.3 | -21.5 |
| Florida | 13.7 | 14.1 | 14.0 | 14.0 | 14.2 | 13.5 | 12.5 | 12.8 | 12.5 | -1.53* | -8.3 |
| Georgia | 16.9 | 15.2 | 15.4 | 15.1 | 15.3 | 14.9 | 16.0 | 15.7 | 15.2 | -0.4 | -9.9 |
| Hawaii | 13.7 | 12.4 | 12.4 | 12.0 | 11.3 | 9.2 | 10.9 | 10.3 | 13.0 | -2.1 | -5.0 |
| Idaho | 12.9 | 13.7 | 13.2 | 12.8 | 12.6 | 13.2 | 13.1 | 12.3 | 14.1 | 0.1 | 8.9 |
| Illinois | 17.2 | 16.2 | 15.7 | 15.5 | 15.6 | 15.4 | 16.6 | 15.1 | 15.8 | -0.8 | -8.0 |
| Indiana | 16.0 | 17.5 | 15.7 | 16.3 | 15.7 | 15.7 | 16.1 | 14.0 | 13.4 | -2.23* | -16.4 |
| Iowa | 15.6 | 14.7 | 14.4 | 13.8 | 15.3 | 14.1 | 16.1 | 15.5 | 14.3 | 0.1 | -7.9 |
| Kansas | 14.4 | 12.4 | 12.1 | 13.2 | 13.1 | 14.7 | 16.4 | 14.4 | 14.4 | 1.9 | -0.1 |
| Kentucky | 16.9 | 16.8 | 14.6 | 15.2 | 14.4 | 14.9 | 15.5 | 15.7 | 14.5 | -1.2 | -14.4 |
| Louisiana | 17.9 | 16.1 | 17.6 | 18.5 | 19.4 | 17.9 | 20.8 | 19.5 | 18.9 | 1.85* | 5.9 |
| Maine | 13.2 | 12.3 | 13.1 | 11.3 | 10.9 | 11.5 | 10.1 | 10.6 | 14.2 | -0.8 | 7.6 |
| Maryland | 16.5 | 17.0 | 16.5 | 16.6 | 17.4 | 15.7 | 15.7 | 16.1 | 17.3 | -0.2 | 5.0 |
| Massachusetts | 14.4 | 14.5 | 13.9 | 14.6 | 14.2 | 13.4 | 12.8 | 12.8 | 13.5 | -1.47* | -6.5 |
| Michigan | 14.8 | 15.8 | 15.8 | 14.5 | 14.2 | 13.1 | 13.7 | 14.3 | 12.8 | -2.04* | -13.2 |
| Minnesota | 12.4 | 11.2 | 12.3 | 12.3 | 13.7 | 11.8 | 13.0 | 12.3 | 13.3 | 1.0 | 7.0 |
| Mississippi | 19.6 | 19.0 | 18.5 | 18.0 | 20.2 | 19.6 | 19.4 | 19.3 | 20.6 | 0.8 | 5.3 |
| Missouri | 16.8 | 16.3 | 15.2 | 14.9 | 15.6 | 16.7 | 17.0 | 14.2 | 15.5 | -0.6 | -7.4 |
| Montana | 13.9 | 11.3 | 10.8 | 12.1 | 12.6 | 13.0 | 13.1 | 12.3 | 9.6 | -1.2 | -30.7 |
| Nebraska | 15.0 | 14.2 | 13.3 | 13.2 | 14.4 | 11.7 | 13.7 | 12.8 | 12.3 | -1.98* | -18.2 |
| Nevada | 12.4 | 10.8 | 12.3 | 12.0 | 11.6 | 11.2 | 11.6 |  |  | -0.8^a^ | -10.6 |
| New Hampshire | 15.6 | 12.9 | 13.2 | 14.7 | 13.4 | 13.2 | 11.9 | 11.9 | 12.0 | -2.67* | -23.0 |
| New Jersey | 13.5 | 13.6 | 13.2 | 13.9 | 13.7 | 13.8 | 13.9 | 13.5 | 13.8 | 0.3 | 2.6 |
| New Mexico | 12.4 | 10.1 | 9.6 | 10.3 | 10.5 | 11.3 | 12.3 | 11.0 | 11.6 | 0.8 | -6.7 |
| New York | 14.9 | 13.2 | 13.7 | 14.1 | 14.0 | 13.9 | 14.3 | 14.6 | 14.4 | 0.4 | -3.2 |
| North Carolina | 18.9 | 19.1 | 17.4 | 18.1 | 17.7 | 17.2 | 17.7 | 16.3 | 16.6 | -1.67* | -12.2 |
| North Dakota | 13.3 | 13.5 | 11.2 | 9.4 | 12.3 | 12.0 | 11.9 | 10.7 | 12.0 | -1.3 | -9.4 |
| Ohio | 15.0 | 14.8 | 14.3 | 14.5 | 15.3 | 14.7 | 15.0 | 15.0 | 15.2 | 0.3 | 1.3 |
| Oklahoma | 14.9 | 15.1 | 13.9 | 14.7 | 14.4 | 15.8 | 14.8 | 15.3 | 13.6 | -0.2 | -8.9 |
| Oregon | 12.9 | 12.6 | 11.2 | 12.4 | 12.1 | 11.7 | 10.6 | 10.9 | 11.3 | -1.84* | -12.3 |
| Pennsylvania | 14.9 | 13.7 | 15.2 | 14.2 | 14.7 | 14.5 | 13.9 | 13.5 | 12.9 | -1.3 | -13.2 |
| Rhode Island | 11.1 | 14.1 | 14.2 | 13.8 | 11.7 | 12.8 | 12.2 | 12.1 | 11.3 | -1.4 | 1.2 |
| South Carolina | 16.5 | 14.1 | 15.3 | 16.1 | 17.8 | 17.5 | 17.0 | 17.2 | 16.7 | 1.4 | 1.1 |
| South Dakota | 16.2 | 11.4 | 16.6 | 12.0 | 12.3 | 7.5 | 11.1 | 12.0 | 12.0 | -4.1 | -25.9 |
| Tennessee | 16.9 | 16.1 | 14.9 | 15.3 | 16.1 | 14.7 | 15.9 | 14.6 | 14.2 | -1.47* | -15.8 |
| Texas | 12.5 | 12.4 | 12.1 | 12.9 | 12.8 | 12.4 | 13.4 | 12.5 | 14.2 | 1.24* | 13.4 |
| Utah | 13.4 | 12.7 | 8.7 | 7.7 | 10.5 | 8.6 | 8.7 | 9.5 | 9.1 | -4.3 | -32.4 |
| Vermont | 11.6 | 15.2 | 11.5 | 16.1 | 12.3 | 12.1 | 9.1 | 12.2 | 11.2 | -2.7 | -4.0 |
| Virginia | 16.8 | 16.2 | 15.8 | 16.1 | 15.8 | 15.7 | 15.5 | 14.9 | 15.4 | -1.10* | -8.4 |
| Washington | 13.8 | 13.9 | 12.1 | 11.4 | 11.9 | 12.4 | 12.2 | 11.8 | 11.8 | -1.77* | -14.8 |
| West Virginia | 15.1 | 14.5 | 15.3 | 13.7 | 16.5 | 14.2 | 13.9 | 15.2 | 14.6 | -0.2 | -3.0 |
| Wisconsin | 15.2 | 14.7 | 13.7 | 13.8 | 13.5 | 13.3 | 14.9 | 14.0 | 14.0 | -0.6 | -8.0 |
| Wyoming | 11.4 | 9.8 | 8.8 | 11.1 | 12.8 | 10.1 | 9.1 | 11.4 | 12.8 | 1.6 | 12.3 |

Abbreviations: APC, annual percentage change; PC, percentage change.

* indicates a significant change.

A temporal trend of TNBC incidence in Nevada was calculated between 2011 and 2017.

Table S4. Joinpoint analysis of trends of TNBC incidence rate by age group in individual races.

| Age group (years) | Trend | APC | 95% CI | *P* value |
| --- | --- | --- | --- | --- |
| NHW |  |  |  |  |
| 0-34 | 2011-2019 | 2.8* | 1.7 - 4.0 | 0.001 |
| 35-49 | 2011-2019 | -1.1* | -1.6 - -0.6 | 0.001 |
| 50-59 | 2011-2019 | -1.0* | -1.6 - -0.4 | 0.006 |
| 60-69 | 2011-2019 | -1.9* | -2.6 - -1.2 | < 0.001 |
| 70 and over | 2011-2019 | 0.1 | -0.6 - 0.7 | 0.811 |
|  |  |  |  |  |
| NHB |  |  |  |  |
| 0-34 | 2011-2019 | -1.2 | -2.6 - 0.2 | 0.081 |
| 35-49 | 2011-2019 | -1.4* | -2.4 - -0.5 | 0.009 |
| 50-59 | 2011-2019 | -0.5 | -1.5 - 0.5 | 0.258 |
| 60-69 | 2011-2019 | -0.8* | -1.5 - -0.2 | 0.021 |
| 70 and over | 2011-2019 | 2.1* | 0.9 -3.2 | 0.004 |
|  |  |  |  |  |
| Hispanic |  |  |  |  |
| 0-34 | 2011-2019 | 4.0* | 1.5 - 6.7 | 0.007 |
| 35-49 | 2011-2019 | -0.1 | -1.5 - 1.4 | 0.911 |
| 50-59 | 2011-2019 | -0.1 | -1.6 - 1.4 | 0.854 |
| 60-69 | 2011-2019 | 0.8 | -0.8 - 2.4 | 0.285 |
| 70 and over | 2011-2019 | 0.7 | -2.3 - 3.9 | 0.597 |
|  |  |  |  |  |
| API |  |  |  |  |
| 0-34 | 2011-2019 | -1.3 | -5.6 - 3.1 | 0.495 |
| 35-49 | 2011-2019 | -1.3 | -3.5 - 1.0 | 0.220 |
| 50-59 | 2011-2019 | 0.7 | -1.6 -3.1 | 0.482 |
| 60-69 | 2011-2019 | 1.0 | -0.7 - 2.9 | 0.211 |
| 70 and over | 2011-2019 | 0.8 | -2.1 - 3.8 | 0.545 |

Abbreviations: AIAN, American Indian/Alaska Native; APC, annual percentage change; API, Asian or Pacific Islander; NHB, Non-Hispanic black; NHW, Non-Hispanic white; TNBC, Triple negative breast cancer.

* indicates a significant change.

Table S5. Joinpoint analysis of trends of TNBC incidence rate by region in individual races.

| Region | Trend 1 | APC | 95% CI | P value | Trend 2 | APC | 95% CI | *P* value |
| --- | --- | --- | --- | --- | --- | --- | --- | --- |
| NHW |  |  |  |  |  |  |  |  |
| Northwest | 2011-2019 | -1.0* | -1.4 - -0.6 | 0.001 |  |  |  |  |
| Midwest | 2011-2019 | -0.8* | -1.7 - -0.02 | 0.047 |  |  |  |  |
| South | 2011-2019 | -0.4 | -0.9 - 0.1 | 0.112 |  |  |  |  |
| West | 2011-2019 | -1.7* | -2.6 - -0.7 | 0.005 |  |  |  |  |
|  |  |  |  |  |  |  |  |  |
| NHB |  |  |  |  |  |  |  |  |
| Northwest | 2011-2019 | 0.3 | -1.3 - 1.9 | 0.671 |  |  |  |  |
| Midwest | 2011-2019 | -0.2 | -0.9 - 0.6 | 0.604 |  |  |  |  |
| South | 2011-2019 | -0.4 | -0.9 - 0.1 | 0.120 |  |  |  |  |
| West | 2011-2019 | -2.1* | -3.2 - -1.0 | 0.003 |  |  |  |  |
|  |  |  |  |  |  |  |  |  |
| Hispanic |  |  |  |  |  |  |  |  |
| Northwest | 2011-2019 | 1.5 | -0.6 - 3.6 | 0.138 |  |  |  |  |
| Midwest | 2011-2019 | -0.0 | -2.2 - 2.2 | 0.997 |  |  |  |  |
| South | 2011-2019 | 0.5 | -0.6 - 1.6 | 0.306 |  |  |  |  |
| West | 2011-2019 | 0.1 | -1.4 - 1.6 | 0.866 |  |  |  |  |
|  |  |  |  |  |  |  |  |  |
| API |  |  |  |  |  |  |  |  |
| Northwest | 2011-2019 | -1.4 | -3.5 - 0.9 | 0.189 |  |  |  |  |
| Midwest | 2011-2019 | -0.1 | -4.4 - 4.5 | 0.971 |  |  |  |  |
| South | 2011-2017 | 2.8* | 0.9 - 4.8 | 0.014 | 2017-2019 | -2.5 | -11.2 - 7.1 | 0.501 |
| West | 2011-2017 | -1.5* | -3.0 - -0.01 | 0.047 | 2017-2019 | 6.9 | -1.5 - 16.0 | 0.086 |

Abbreviations: AIAN, American Indian/Alaska Native; APC, annual percentage change; API, Asian or Pacific Islander; NHB, Non-Hispanic black; NHW, Non-Hispanic white; TNBC, Triple negative breast cancer.

* indicates a significant change.

Table S6. Joinpoint analysis of trends of TNBC incidence rate by disease stage in individual races.

| Disease stage | Trend | APC | 95% CI | *P* value |
| --- | --- | --- | --- | --- |
| NHW |  |  |  |  |
| Localized | 2011-2019 | -0.5* | -1.0 - -0.1 | 0.033 |
| Regional | 2011-2019 | -0.8* | -1.3 - -0.4 | 0.004 |
| Distant | 2011-2019 | 0.04 | -0.9 - 1.0 | 0.923 |
|  |  |  |  |  |
| NHB |  |  |  |  |
| Localized | 2011-2019 | 0.1 | -0.6 - 0.7 | 0.818 |
| Regional | 2011-2019 | -0.5 | -1.1 - 0.1 | 0.098 |
| Distant | 2011-2019 | 0.5 | -0.4 - 1.4 | 0.265 |
|  |  |  |  |  |
| Hispanic |  |  |  |  |
| Localized | 2011-2019 | 0.8 | -0.3 - 1.9 | 0.134 |
| Regional | 2011-2019 | 0.5 | -0.5 - 1.5 | 0.283 |
| Distant | 2011-2019 | 1.3 | -1.0 - 3.7 | 0.220 |
|  |  |  |  |  |
| API |  |  |  |  |
| Localized | 2011-2019 | 0.5 | -1.3 - 2.3 | 0.541 |
| Regional | 2011-2019 | -0.1 | -1.5 - 1.3 | 0.831 |
| Distant | 2011-2019 | 2.0 | -2.9 - 7.2 | 0.368 |

Abbreviations: AIAN, American Indian/Alaska Native; APC, annual percentage change; API, Asian or Pacific Islander; NHB, Non-Hispanic black; NHW, Non-Hispanic white; TNBC, Triple negative breast cancer.

* indicates a significant change.
